# Long-Term Changes in Daily Stress Reactivity as a Mediator Between Changes in Health and Well-Being Across 20 Years

**DOI:** 10.1093/geroni/igaf122.824

**Published:** 2025-12-31

**Authors:** Jonathan Rush, Eric Cerino, Dakota Witzel, Jennifer Piazza, Susan Charles, David Almeida

**Affiliations:** University of Victoria, Victoria, British Columbia, Canada; Northern Arizona University, Flagstaff, Arizona, United States; South Dakota State University, Brookings, South Dakota, United States; California State University, Fullerton, Fullerton, California, United States; University of California, Irvine, Irvine, California, United States; The Pennsylvania State University, University Park, Pennsylvania, United States

## Abstract

The daily within-person association between stress exposure and negative affect (i.e., stress reactivity) has been shown to be predictive of adverse health and well-being outcomes. These findings typically rely on a single burst of daily assessments. Recent findings have shown that long-term changes in daily stress reactivity across adulthood are associated with changes in health outcomes. The present study extends this work by examining whether long-term changes in daily stress reactivity mediates the association between longitudinal changes in functional health and life satisfaction across 20 years. We used measurement burst data from the National Study of Daily Experiences (N = 2,880) embedded within the MIDUS longitudinal study. Three measurement bursts were separated by ten years, with each containing daily measures of stress and affect across eight consecutive days, yielding 33,942 days of data across 20 years of adulthood. Functional health and life satisfaction were also measured every 10 years. Multilevel SEM were fit to simultaneously model daily within-person associations between stress and affect (i.e., stress reactivity) at Level 1; long-term changes in stress reactivity at Level 2; and the mediational effect of changes in stress reactivity on the pathway of changes in functional health predicting changes in life satisfaction at Level 3. Declines in functional health predicted declines in life satisfaction across 20 years (estimate=1.12, SE = 0.37, p<.001). Importantly, changes in stress reactivity mediated this effect (indirect effect=17.51, SE = 8.40, p=.02). Results suggest that the link between declines in functional health and life satisfaction may be accounted for by increases in daily stress reactivity.

